# Association between *CC10 *+38A/G polymorphism and asthma risk: A meta-analysis

**DOI:** 10.12669/pjms.296.3724

**Published:** 2013

**Authors:** Guangri Zhao, Xiaodan Lin, Ming Zhou, Jian Zhao

**Affiliations:** 1 Guangri Zhao, Department of Chest Surgery, Department of Chest Surgery, Guangzhou Medical University Cancer Institute and Hospital, Guangzhou, China.; 2 Xiaodan Lin, Department of Radiotherapy, Guangzhou Medical University Cancer Institute and Hospital, Guangzhou, China.; 3 Ming Zhou, Department of Chest Surgery, Guangzhou Medical University Cancer Institute and Hospital, Guangzhou, China.; 4 Jian Zhao, Guangzhou Medical University Cancer Institute and Hospital, Guangzhou, China.

**Keywords:** Asthma, CC10, Meta-analysis, Polymorphism

## Abstract

***Objectives: ***A number of studies conducted to assess the association between *Clara cell 10-kDa protein (CC10) *+38A/G polymorphism and susceptibility to asthma have yielded inconsistent and inconclusive results. In the present study, the possible association was assessed by a meta-analysis.

***Methods: ***Relevant articles were identified for the period ranging from Jan 1998 up to March 2013. Pooled odds ratios (OR) with 95% confidence intervals (CI) were appropriately derived from fixed effects or random-effects models.

***Results: ***Ten case-control studies with a total of 1529 asthma cases and 2399 controls were included in this meta-analysis. The association between *CC10 *+38A/G polymorphism and asthma risk was determined in dominant model, recessive model, additive model, and codominant model. In dominant model, *CC10 *+38A/G polymorphism seemed to be associated with elevated asthma risk (OR = 1.62; 95% CI, 1.23-2.12; *P *= 0.0005). Subgroup analyses by ethnicity also found significant associations between this polymorphism and asthma risk in Asians and Caucasians. Results from other genetic models further identified this possible association.

***Conclusion: ***This meta-analysis suggests that *CC10 *+38A/G polymorphism confers asthma risk.

## INTRODUCTION

Asthma is an inflammatory disorder of the airways characterized by reversible airway obstruction and bronchial hyper-responsiveness. Although environmental factors are important determinants of asthma, numerous studies have revealed that asthma has a strong genetic component.^1^

Clara cell 10-kDa protein (CC10, also known as CC16, SCGB1A1, uteroglobin) is a 10-kDa protein produced by non-ciliated Clara cells and is one of the most abundant proteins in the fluids lining the airways.^2^ The *CC10* gene is located on chromosome 11q12-13, a region that has been associated with asthma and atopy in several genome-wide linkage studies.^3^^,^^4^ A single nucleotide polymorphism, the +38A/G polymorphism (rs3741240), has been identified in this gene.^5^ Some papers have reported that this polymorphism was associated with asthma risk, whereas others found no such association.^5^^-^^14^ It was possible that most of these studies only included a modest sample size, and thus each of them might not achieve a reliable conclusion. Meta-analysis is a useful method for investigating associations between genetic factors and diseases, because a quantitative approach is used to combine the results from different studies on the same topic, thereby providing more reliable conclusions.^15^ Here, we use meta-analysis to clarify the association of *CC10 *+38A/G polymorphism with asthma. To our knowledge, this is the first genetic meta-analysis of the association between *CC10 *+38A/G polymorphism and the risk of asthma.

## METHODS

***Search for publications:*** We performed a literature search using the MEDLINE and China National Knowledge Infrastructure (CNKI) databases to identify articles that examined associations between *CC10 *+38A/G polymorphism and asthma. The following key words and subject terms were searched: Clara cell 10-kDa protein, CC10, CC16, SCGB1A1, uteroglobin, asthma, genetic, and polymorphism. Reference lists of articles retained for review were inspected for relevant publications. No publication date or language restrictions were imposed.

***Inclusion and exclusion criteria:*** The following inclusion criteria were used: (i) the study should have evaluated the association between the +38A/G polymorphism and asthma risk; (ii) the study should have had a case-control design; (iii) sufficient data should have been provided in order to calculate odds ratios (OR) and 95% confidence intervals (CI). Studies were excluded if any of the following conditions applied: (i) only abstracts or reviews were available, without sufficient data; (ii) genotype frequencies were not reported; (iii) studies were repeated or publications overlapped.

***Data extraction:*** The following information was extracted from each study: author, year of publication, ethnicity of the study population, numbers of cases and controls, and *CC10 *+38A/G genotype numbers.

***Statistical analysis:*** Meta-analyses were performed using dominant model (AA + AG vs GG). Heterogeneity among studies was assessed using the chi-square based *Q* and *I*^2^ statistics. Heterogeneity was considered to be significant for *I*^2^ > 50%. Fixed-effects or random-effects models were used to pool the data according to heterogeneity. Other comparative genetic models were also used to assess the association between the polymorphism and the risk of asthma (AA vs AG + GG, AA vs GG, AG vs GG, and A vs G). For the subgroup analysis by ethnicity, the study populations were stratified into two groups: Asian and Caucasian. Galbraith plot was used to spot the outliers which were the sources of heterogeneity. Funnel plot and Egger’s test were used to detect publication bias.^16^ Analyses were performed using Revman 5.1 and STATA 11.0 software.

## RESULTS

***Characteristics of studies:*** A total of 10 relevant articles on *CC10 *+38A/G polymorphism in asthma met the study inclusion criteria (Table-I), and were included in the meta-analysis.^5^^-^^14^ Finally, 1529 genotyped asthma cases and 2399 genotyped non-asthmatic controls were included in the meta-analysis. Five case-control studies were performed in Asian populations; while five were performed in Caucasian populations. The characteristics of each case-control study and the genotype and allele distributions in each case-control study are presented in Table-I.

**Fig.1:**
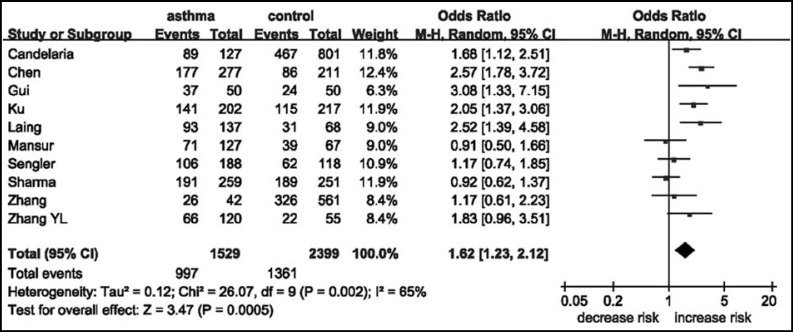
Meta-analysis of the correlation between the *CC10* +38A/G polymorphism and asthma risk using a dominant model

**Fig.2:**
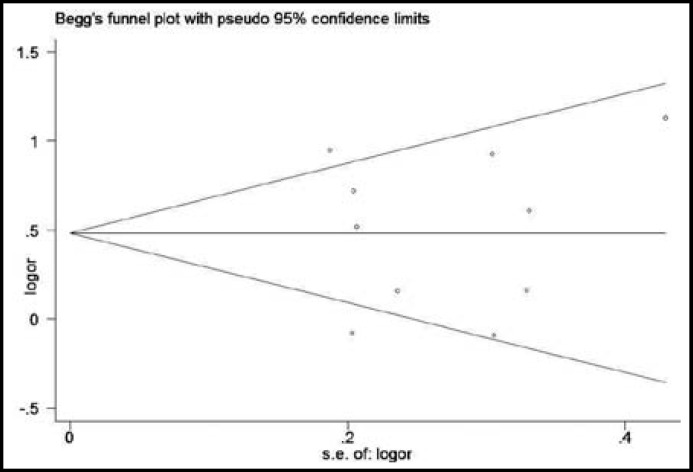
Begg’s funnel plot for evaluation of publication bias in the selection of studies on the association between asthma risk and the *CC10* +38A/G polymorphism (dominant model).

**Fig.3:**
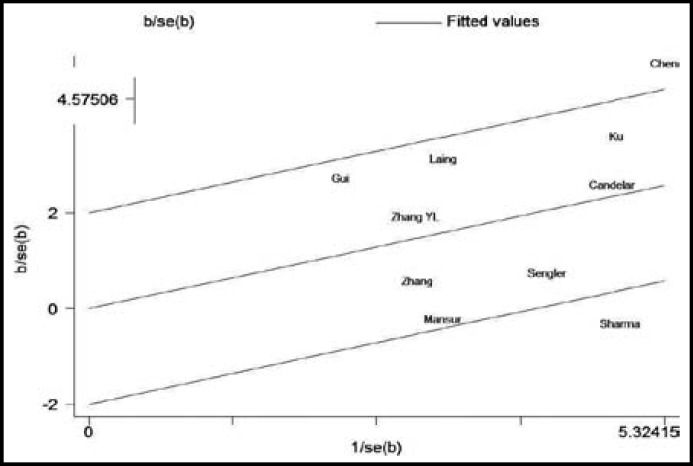
Galbraith plots of association between *CC10* +38A/G polymorphism and asthma risk

**Table-I:** Characteristics of the individual studies included in the meta-analysis

			*Cases/*	*Asthma genotypes*				*Asthma genotypes*		
*Author (Ref)*	*Year*	*Ethnicity*	*Controls (n)*	*AA*	*AG*	*GG*		*AA*	*AG*	*GG*
Laing (5)	1998	Caucasian	137/68	26	67	44		5	26	37
Mansur (6)	2002	Caucasian	127/67	14	57	56		8	31	28
Sengler (7)	2003	Caucasian	188/118	15	91	82		12	50	56
Gui (8)	2003	Asian	50/50	16	21	13		8	16	26
Sharma (9)	2004	Asian	259/251	72	119	68		74	115	62
Candelaria (10)	2005	Caucasian	127/801	23	66	38		81	386	334
Zhang (11)	2009	Caucasian	42/561	5	21	16		77	249	235
Ku (12)	2011	Asian	202/217	63	78	61		33	82	102
Chen (13)	2012	Asian	277/211	177*		100		86*		125
Zhang YL (14)	2012	Asian	120/55	19	47	54		5	17	33

* number of AA genotype and AG genotype. NA, not available

**Table-II:** Summary of comparisons based on different genotypes

		*Sample size*		*No. of*	*Test of association*		
	*Study*	*case*	*control*	*studies*	*OR (95% CI)*	*Z*	*P value*	
AA + AG vs. GG	Overall	1529	2399	10	1.62 (1.23 – 2.12)	3.47	0.0005	
	Asian	908	784	5	1.87 (1.20 – 2.91)	2.78	0.005	
	Caucasian	621	1615	5	1.41 (1.02 – 1.94)	2.08	0.04	
AA vs. AG + GG	Overall	1252	2188	9	1.48 (1.02 – 2.15)	2.09	0.04	
	Asian	631	573	4	1.42 (0.92 – 3.26)	1.69	0.09	
	Caucasian	621	1615	5	1.31 (0.79 – 2.18)	1.05	0.29	
AA vs. GG	Overall	685	1216	9	1.77 (1.11 – 2.82)	2.41	0.02	
	Asian	366	343	4	2.14 (0.96 – 4.81)	1.85	0.06	
	Caucasian	319	873	5	1.53 (0.82 – 2.86)	1.33	0.18	
AG vs. GG	Overall	999	1885	9	1.37 (1.14 – 1.64)	3.43	0.0006	
	Asian	461	453	4	1.36 (1.04 – 1.78)	2.26	0.02	
	Caucasian	538	1432	5	1.37 (1.08 – 1.75)	2.58	0.01	
A vs. G	Overall	2504	4376	9	1.40 (1.10 – 1.78)	2.72	0.006	
	Asian	1262	1146	4	1.60 (1.00 – 2.57)	1.97	0.05	
	Caucasian	1242	3230	5	1.27 (0.96 – 1.68)	1.70	0.09	

***Results of meta-analyses:*** As shown in Fig.1, the heterogeneity of AA + AG vs GG was assessed for all 10 studies, and the chi-square value was 26.07 with 9 degrees of freedom and *P *= 0.002 in a random-effects model. Therefore, a random-effects model was used for synthesis of the data. The overall OR was 1.62 (95% CI, 1.23-2.12), and the *Z*-test value for overall effect was 3.47 (*P *= 0.0005) for the AA + AG vs GG. Subgroup analysis by ethnicity was performed. For ethnicity, the populations were stratified into two groups: Asian (908 cases and 784 controls), Caucasian (621 cases and 1615 controls). There were significant associations with asthma risk in these populations: Asian (OR = 1.87; 95% CI, 1.20-2.91; *P *= 0.005) and Caucasian (OR = 1.41; 95% CI, 1.02-1.94; *P *= 0.04). Publication bias was assessed graphically by using funnel plots and assessed statistically by using Egger’s test. The shape of the Begg’s funnel plots appeared symmetrical for the AA + AG vs GG model (Fig.2). Egger’s test was performed to provide statistical evidence of funnel plot asymmetry (*P *= 0.99). Summary results from the other comparisons are presented in Table-II.

***Heterogeneity analysis:*** The between-study heterogeneity was obvious in dominant model (*I*^2^ = 65%). Galbraith plots spotted Sharma’s study and Chen’s study as the outliers and the major source of the heterogeneity (Fig.3). The heterogeneity remarkably decreased in dominant model after excluding Sharma’s study and Chen’s study (*I*^2^ = 43%). Besides, a significant association was also observed after excluding Sharma’s study and Chen’s study (OR = 1.63; 95% CI, 1.26-2.11; *P *= 0.0002).

## DISCUSSION

CC10 with primary expression in the uterus and non-ciliated bronchiolar cells, has an anti-inflammatory effect on the urogenital and respiratory tracts.^17^^,^^18^ Deficiency of *CC10* aggravates pulmonary allergic inflammation through augmentation of the TH2 response, and reconstitution of CC10 in *CC10*-deficient mice is able to reverse the altered phenotypes.^19^ These results suggested that CC10 played an important role in the development of asthma. Although asthma can have many causes, genetic factors are considered strong determinants, and therefore, researchers have searched for the genes responsible. Several studies have investigated the role of *CC10* +38A/G polymorphism in asthma susceptibility both in Asians and Caucasians, with contrasting results. Meta-analysis has been recognized as an important tool to more precisely define the effect of selected genetic polymorphisms on risk of disease. Therefore, we performed the meta-analysis specifically to assess this association.

The present meta-analysis found a significant association between the *CC10* +38A/G polymorphism and asthma risk (OR = 1.62; 95% CI, 1.23-2.12; *P *= 0.0005). In addition, subgroup analysis by ethnicity showed that this polymorphism was significantly associated with increased asthma risk in both the Caucasians (OR = 1.41; 95% CI, 1.02-1.94; *P *= 0.04) and Asians (OR = 1.87; 95% CI, 1.20-2.91; *P *= 0.005). Moreover, other genetic models also found positive results. Thus, meta-analysis of available data suggests *CC10* +38A allele contributes to increased asthma risk.

The importance of heterogeneity should be mentioned, as it is an important issue when interpreting the results of meta-analyses. In this present meta-analysis, we found obvious heterogeneity (*I*^2 ^= 65%). Galbraith plot was used to spot the outliers as the possible major sources of heterogeneity. Two studies were spotted as the outliers. After excluding those two studies, the between-study heterogeneity decreased and there was no obvious heterogeneity among the left 8 studies (*I*^2 ^= 43%), which suggested the heterogeneity might come from those two studies. Meta-analysis of the left 8 studies also showed *CC10* +38A/G polymorphism was associated with increased risk of asthma (OR = 1.63; 95% CI,1.26-2.11; *P *= 0.0002).

Some possible limitations in this meta-analysis should be acknowledged. First, only published studies that were included in the selected electronic databases were identified; it is possible that some relevant published or unpublished studies that supported the null hypothesis may have been missed, which may have biased the results, and this may not have been detected by the statistical tests that were performed. Second, the effect of gene-gene and gene-environment interactions was not addressed in this meta-analysis status, because of limited available data. Third, all the data were used without adjustment by detailed individual information such as age, sex, and lifestyle in our meta-analysis. Finally, the number of included studies was moderate.

To sum up, this meta-analysis suggests that *CC10* +38A/G polymorphism confers asthma risk. Further studies can assess the possible gene-environmental and gene-gene interactions in the association between this polymorphism and asthma risk.

## Author Contribution:

**Guangri Zhao, Xiaodan Lin, Ming Zhou and Jian Zhao: **All the authors had** s**ubstantial contributions to conception and design, acquisition of data, analysis and interpretation of data, drafting the manuscript and final approval of the version to be published.
